# PSMD14 stabilizes estrogen signaling and facilitates breast cancer progression via deubiquitinating ERα

**DOI:** 10.1038/s41388-023-02905-1

**Published:** 2023-11-29

**Authors:** Penghe Yang, Xiao Yang, Dehai Wang, Huijie Yang, Zhongbo Li, Chenmiao Zhang, Shuqing Zhang, Jian Zhu, Xin Li, Peng Su, Ting Zhuang

**Affiliations:** 1 Xinxiang Key Laboratory of Tumor Migration and Invasion Precision Medicine, School of Medical Technology, Xinxiang Medical University, Xinxiang, 453003 Henan Province PR China; 2 Henan Key Laboratory of Immunology and Targeted Therapy, School of Medical Technology, Xinxiang Medical University, Xinxiang, 453003 Henan Province PR China; 3 Department of Laboratory Medicine, Xinxiang Central Hospital, Xinxiang, 453003 Henan Province PR China; 4 Department of General Surgery, Shengjing Hospital of China Medical University, Shenyang, 110000 Liaoning Province PR China; 5 Department of General Surgery, The Second Hospital, Cheeloo College of Medicine, Shandong University, Jinan, 250033 Shandong Province PR China; 6 Department of Surgical Oncology and General Surgery, The First Hospital of China Medical University, Shenyang, 110000 Liaoning Province PR China; 7 Department of Pathology, Qilu Hospital, Cheeloo College of Medicine, Shandong University, Jinan, 250012 Shandong Province PR China

**Keywords:** Breast cancer, Nuclear receptors

## Abstract

The over-activation of ERα signaling is regarded as the major driver for luminal breast cancers, which could be effective controlled via selective estrogen receptor modulators (SERM), such as tamoxifen. The endocrine resistance is still a challenge for breast cancer treatment, while recently studies implicate the post-translational modification on ERα play important roles in endocrine resistance. The stability of ERα protein and ERα transcriptome are subject to a balance between E3 ubiquitin ligases and deubiquitinases. Through deubiquitinases siRNA library screening, we discover PSMD14 as a critical deubiquitinase for ERα signaling and breast cancer progression. PSMD14 could facilitate breast cancer progression through ERα signaling in vitro and in vivo, while pharmaceutical inhibition of PSMD14 via Thiolutin could block the tumorigenesis in breast cancer. In endocrine resistant models, PSMD14 inhibition could de-stabilize the resistant form of ERα (Y537S) and restore tamoxifen sensitivity. Molecular studies reveal that PSMD14 could inhibition K48-linked poly-ubiquitination on ERα, facilitate ERα transcriptome. Interestingly, ChIP assay shows that ERα could bind to the promoter region of PSMD14 and facilitate its gene transcription, which indicates PSMD14 is both the upstream modulator and downstream target for ERα signaling in breast cancer. In general, we identified a novel positive feedback loop between PSMD14 and ERα signaling in breast cancer progression, while blockade of PSMD14 could be a plausible strategy for luminal breast cancer.

## Background

According to recent world cancer statistics, breast cancer accounts for 24% of women malignancies and 15% of cancer-related deaths in females [1]. According to molecular pathological classification, breast cancer is composed of Luminal A type, Luminal B type, HER2 type and TNBC (Triple negative breast cancer) [2]. Both Luminal A and B type breast cancers are ERα positive, which could be well controlled via endocrine therapy [3]. However, the emerge of endocrine resistance will eventually happen in more than 50% of breast cancer patients, making it an urgent clinical issue [4]. Several cancer biology studies proposed quite a few regulation models to explain endocrine resistance, such as transformation into ERα negative and the ERα constitutive active mutations in certain functional domains [5]. Interestingly, more than half of endocrine resistance breast cancers remain ERα positive [6]. Mechanisms of endocrine resistance remain unclear to cancer biology researchers.

The logical link between estrogen signaling and breast cancer has been established for more than 80 years [7]. The hormone receptor ERα was firstly cloned in 1985, which is the major driver for the oncogenic process in luminal type breast cancers [8]. ERα protein is composed of 595 amino acids including trans-activation domain 1 (AF1), Ligand Binding Domain (LBD) and DNA binding domain (DBD) [9]. The DNA binding domain could interact with the estrogen response elements in DNA and facilitate ERα target gene transcription, while the LBD domain is responsible for the interaction with estradiol and tamoxifen [10]. When ERα is activated, it trans-locates into the nuclear and trans-activates the target gene expression, including TFF1 and GREB1 [11]. Subsequently the activation of ERα promotes breast cancer cell proliferation and progression [12]. Tamoxifen, which shares similar structure with estradiol, functions to compete with estrogen for DNA binding and block ERα target gene expression [13]. Since the endocrine resistance becomes the major challenge for breast cancer patients, the understanding of ERα signaling activity, including ERα expression and stability regulation, is important in the development of novel anti-estrogen therapy and overcome endocrine resistance.

The ubiquitin-proteasome system (UPS) plays a pivotal role in the regulation of protein stability and degradation [14], while the protein ubiquitination process is a reversible process, which is a cascade including E1 ligases, E2 ligases and E3 ligases. The ubiquitination process is counter-balanced by deubiquitination [15]. According to the current knowledge, there are about 100 deubiquitinases coded in human genome, which is composed of UCH family deubiquitinases, USP family deubiquitinases, OUT deubiquitinases, Josephin family deubiquitinases and JAMM family deubiquitinases [16, 17]. Recent studies revealed that a few deubiquitinases could stabilize ERα and promote breast cancer progression [18, 19]. However, since there are about 100 deubiquitinases in human, which of the deubiquitinases are critical in estrogen signaling and breast cancer growth is still not clear. PSMD14 (Proteasome 26S Subunit, Non-ATPase 14; Rpn11; POH1) belongs to the JAMM superfamily of deubiquitinases [20]. PSMD14 could specially cleave the ubiquitin chains from the substrates and therefore play important role in cellular hemostasis [16]. Quite a few studies demonstrated that PSMD14 could participate several cellular activities, including DNA damage response, genomic transcription and cell senescence [21–23]. The expression of PSMD14 was reported to correlate with poor overall survival in several human malignancies, such as liver cancer and esophageal cancer [24, 25]. For example, PSMD14 could deubiquitinase and stabilize TGF-beta, which facilitates liver cancer progression [26]. Besides, PSMD14 could associate with snail, which inhibits snail poly-ubiquitination and degradation in esophageal cancer [27]. However, the regulation of PSMD14 in estrogen signaling remains unclear in breast cancer. In our study, our aim is to identify key deubiquitinases that significantly impact breast cancer progression and have important implications for breast cancer therapeutics.

## Results

### PSMD14 is elevated in human breast cancer and correlates with poor survival in luminal type breast cancer

**Fig. 1:**
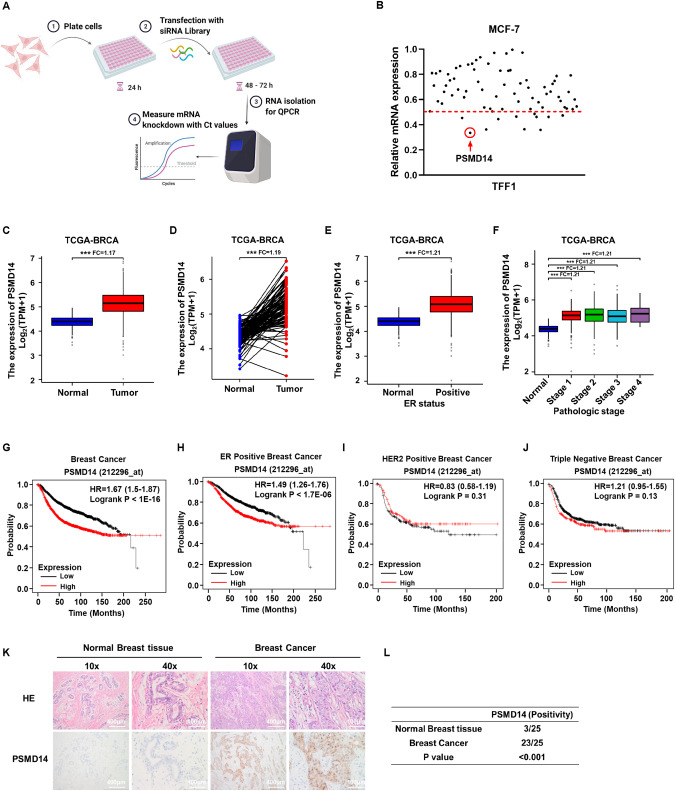
PSMD14 is a candidate DUB of ERα that correlates with poor prognosis in ER positive breast cancer. **A** Diagram of the screening procedure used to identify ERα DUBs. Each of the 76 human DUB genes was knocked down in MCF-7 cells with 20uM pooled siRNAs. After 48 h, the quantitative gene expression analysis was detected by Real-time PCR. **B** The classical target gene TFF1 was used to indicate ERα. The siRNA screening data showed that PSMD14 was required for TFF1 gene expression in MCF-7 cells. **C**, **D** PSMD14 expression level was significantly elevated in breast cancer tissues compared to normal tissues from TCGA database (https://www.genome.gov/). **E** The PSMD14 expression level was significantly elevated in ER positive breast cancer tissues compared with normal breast tissue. Data were generated from the TCGA database (https://www.genome.gov/). **F** The PSMD14 expression level was significantly elevated in breast cancer tissues of different stages compared with normal breast tissue. Data were generated from the TCGA database (https://www.genome.gov/). **G**–**J** Kaplan−Meier analysis showing relapse-free survival depending on PSMD14 expression levels from public meta-analysis data (https://kmplot.com). PSMD14 expression was correlated with poor survival in ERα positive human breast cancer but not in HER2 positive and triple negative breast cancer. P values were calculated using log-rank test. **K**, **L** Immunohistochemical staining showed that the expression of PSMD14 protein is significantly increased in breast cancer compared with normal breast tissue. Statistical analysis of PSMD14 expression in 25 breast cancer samples. All P values were calculated by unpaired two-tailed Student’s t tests. *P < 0.05; **P < 0.01; ***P < 0.001.

Since the aim of the study was to identifying novel DUBs in regulation estrogen signaling, we did the siRNA screening using the DUBs siRNA library (Dharmacon Company, Cat: G104705) in MCF-7 cells (Fig. 1A). As TFF1 was recognized as the most classical ERα target genes, we utilized TFF1 as the indicator in estrogen signaling activity. The data showed that PSMD14 depletion significantly inhibited TFF1 expression in MCF-7 cells (Fig. 1B). We further investigated PSMD14 expression in human breast cancer, which showed that PSMD14 was elevated in breast malignancies from TCGA database (Fig. 1C, D). Besides, PSMD14 was also increased in ERα positive breast cancer samples (Fig. 1E). The subgroup analysis showed that PSMD14 was increased in all subtype of breast cancer samples and all stages of breast cancers (Fig. 1F). We further explored the impact of PSMD14 in breast cancer survival from kmplot database (https://kmplot.com/analysis/). The survival data showed that PSMD14 correlated with poor survival in all breast cancer patients (Fig. 1G). The subgroup analysis showed that PSMD14 correlated with poor survival only in ER positive breast cancer patients, but not in HER2 positive and TNBC types, which could indicate the survival impact of PSMD14 is dependent on ER status (Fig. 1H–J). We further examined the protein level of PSMD14 in breast cancer patient samples through immunohistochemistry (IHC), while PSMD14 expression was significantly increased in breast cancers (3/25 vs 23/25; *P* < 0.001; Fig. 1K, L).

### PSMD14 is required for ERα positive breast cancer progression

**Fig. 2:**
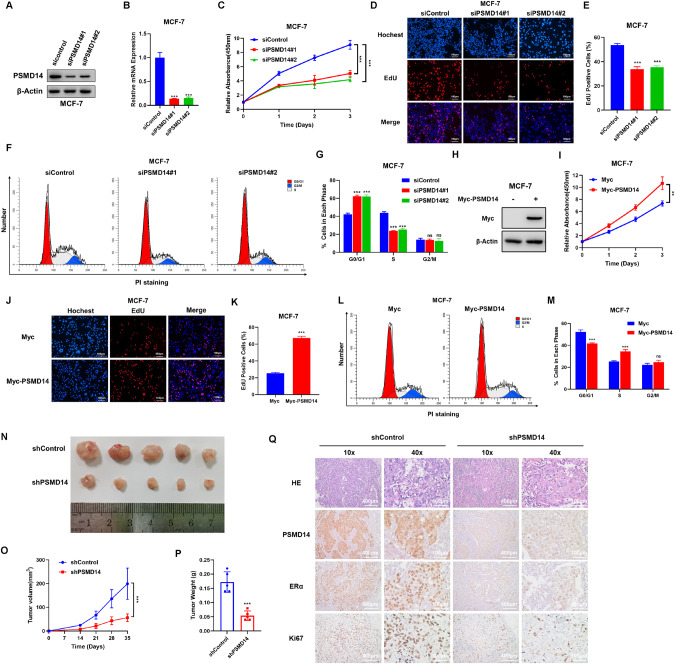
PSMD14 depletion inhibits cell proliferation in ER positive breast cancer. **A**, **B** Immunoblot analysis and qRT-PCR showing the expression level of PSMD14 in MCF-7 cells transfected with siControl or two independent siPSMD14. β-Actin was used as the internal control. **C** PSMD14 depletion inhibits the proliferation of ER positive breast cancer cells. MCF-7 cells were transfected with 50 nM siControl or 50 nM PSMD14. After 24 h, a CCK-8 assay was used to determine the cellular metabolic activity at the indicated time points after transfection. Experiments were performed in triplicate. *P < 0.05; **P < 0.01; ***P < 0.001 for cell growth comparisons. **D**, **E** PSMD14 depletion reduced the number of EdU-positive ER positive breast cancer cells. MCF-7 cells were transfected with 50 nM siControl or 50 nM PSMD14. After 24 h, EdU was added to the medium for 2 h of incubation. The absolute cell number was determined to indicate cell proliferation activity (**D**). Right panel shows quantification of EdU results by ImageJ software (**E**). Scale bar 100 μm. *N* = 3, *P < 0.05; **P < 0.01; ***P < 0.001 for cell growth comparisons. **F**, **G** Cell-cycle analysis by flow cytometry of MCF-7 cells transfected with 50 nM siControl or 50 nM PSMD14. After 24 h, the cells were harvested, fixed with 70% ethanol, and stained with propidium iodide. The cells were subjected to FACS analysis. Experiments were performed in triplicate. *P < 0.05; **P < 0.01; ***P < 0.001 for cell proportion comparisons. **H** The immunoblot analysis indicated the expression level of PSMD14 in MCF-7 cells transfected with either Myc or Myc-PSMD14, using β-Actin as the internal control. **I** PSMD14 promotes proliferation in MCF-7 cells. The cell proliferation rate was acquired by CCK-8 assay. MCF-7 cells were transfected with 1 μg Myc vector or Myc-PSMD14. After 24 h, a CCK-8 assay was used to determine the cellular metabolic activity at the indicated time points after transfection. Experiments were performed in triplicate. *P < 0.05; **P < 0.01; ***P < 0.001 for cell growth comparisons. **J**, **K** PSMD14 promotes the number of EdU-positive ER POSITIVE breast cancer cells. MCF-7 cells were transfected with 1 μg Myc vector or Myc-PSMD14. After 24 h, EdU was added to the medium for 2 h of incubation. The absolute cell number was determined to indicate cell proliferation activity. Right panel shows quantification of Edu results by ImageJ software. Scale bar 100 μm. *N* = 3, *P < 0.05; **P < 0.01; ***P < 0.001 for cell growth comparisons. **L**, **M** Cell-cycle analysis by flow cytometry of MCF-7 cells transfected with 1 μg Myc vector or Myc-PSMD14. After 24 h, the cells were harvested, fixed with 70% ethanol, and stained with propidium iodide. The cells were subjected to FACS analysis. Experiments were performed in triplicate. *P < 0.05; **P < 0.01; ***P < 0.001 for cell proportion comparisons. PSMD14 depletion inhibits the tumor growth of T47D cells in a xenograft model. Harvested and photographed tumors in the shPSMD14 and the shControl (**N**), tumor volume (**O**) and weight (**P**) growth in each mouse from the shPSMD14 group and the Control group in vivo. *P < 0.05; **P < 0.01; ***P < 0.001. **Q** The levels of PSMD14, ERα and Ki67 in xenografts by IHC staining. Scale bar, 100 µm.

We conducted further investigations on the role of PSMD14 in MCF-7, T47D and MDA-MB-175 cells. Depleting PSMD14 in these cells resulted in a significant effect (Fig. 2A, B, Fig S1A–D). The CCK8 assay revealed that PSMD14 depletion inhibited cancer cell proliferation in MCF-7, T47D and MDA-MB-175 cells (Fig. 2C, Fig S1E, F). Furthermore, the EdU incorporation assay demonstrated that PSMD14 depletion reduced the number of EdU positive cells in MCF-7, T47D and MDA-MB-175 (Fig. 2D, E, Fig S1G–J). In addition, flow cytometry analysis of the cell cycle showed that PSMD14 depletion led to a significant increase in the proportion of cells in the G0/G1 phase in MCF-7, T47D and MDA-MB-175 (Fig. 2F, G, Fig S1K–N). To explore the effect of PSMD14 overexpression, we created a stable cell line with PSMD14 overexpression (Fig. 2H). The CCK8 assay revealed that overexpression of PSMD14 promoted cancer cell proliferation in MCF-7 cells (Fig. 2I). The EdU incorporation assay revealed that PSMD14 overexpression increased the number of EdU positive cells in MCF-7 cells (Fig. 2J, K). Furthermore, flow cytometry analysis showed that PSMD14 overexpression significantly increased the proportion of cells in the S phase in MCF-7 cells (Fig. 2L, M). Additionally, our findings were confirmed in xenograft models, with PSMD14 depletion effectively inhibiting ERα positive tumor growth in vivo. Immunohistochemistry analysis revealed decreased expression of Ki67 and ERα in PSMD14 depletion tumors (Fig. 2N–Q).

### Global gene expression analysis shows PSMD14 correlates with ERα signaling activity in breast cancer

**Fig. 3:**
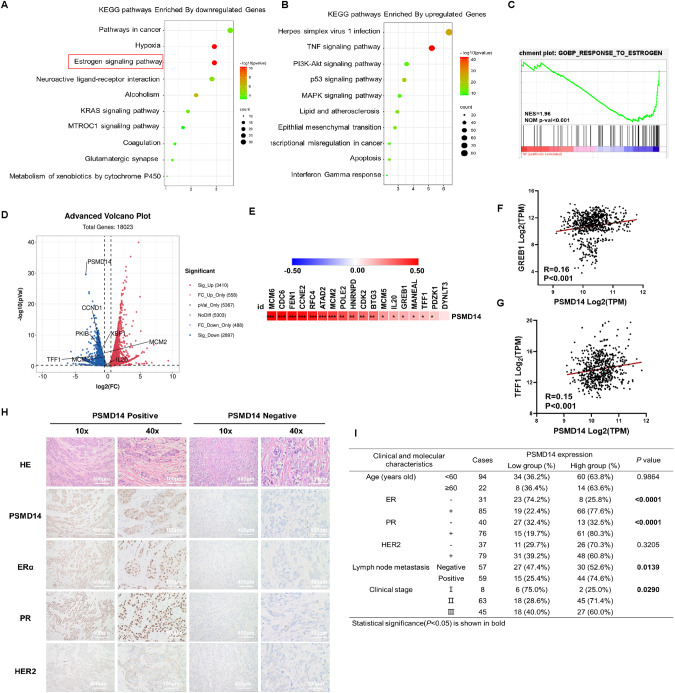
Bioinformatic analysis reveals PSMD14 correlates with ERα signaling activity in breast cancer. **A**, **B** The PSMD14 was silenced in MCF-7 cells, and total RNA was extracted after 48 h. RNA sequencing was performed on total RNA samples from the siControl and siPSMD14 groups. KEGG analysis was performed on the RNA sequencing data to identify the estrogen signaling pathway regulated by PSMD14. **C** Gene set enrichment analysis (GSEA) plots showing the enrichment of genes associated with estrogen signaling (lower) in the siPSMD14 group of the RNA-sequencing data. **D** Volcano plot of RNA sequencing data demonstrating the expression changes of several ERα target genes upon siControl or siPSMD14. **E**–**G** Publicly available data demonstrating that PSMD14 expression is correlated with that of the ERα target genes. (https://tcga-data.nci.nih.gov/tcga/). **H**, **I** Correlations between the PSMD14 expression level in breast tumor samples and clinicopathological characteristics of the corresponding patients. Data analysis revealed that PSMD14 expression was correlated with ER status (*P* < 0.0001), PR status (*P* < 0.0001) and lymph node metastasis (*P* = 0.0139) and advanced tumor stage (*P* = 0.0290).

We further analyzed the effect of PSMD14 depletion in MCF-7 cells by conducting whole genomic expression profiling. RNA sequence analysis was performed and the data sent to Beijing Genomics institution for analysis. Our findings indicate that PSMD14 depletion inhibited several oncogenic pathways, such as the estrogen signaling pathway and KRAS pathway. Simultaneously, we observed the activation of several tumor suppression pathways including the P53 and apoptosis pathways (Fig. 3A, B). Our analysis using Gene Signature Enrichment Analysis (GSEA) demonstrated that PSMD14 depletion globally inhibited estrogen signaling activity (Fig. 3C). Moreover, volcano plot revealed that PSMD14 depletion effectively suppressed the expression of classical ERα target genes, such as TFF1, PKIB, and CCND1 (Fig. 3D). Furthermore, we investigated the correlation between PSMD14 and classical ERα target genes in breast tumors from the TCGA database. The results indicated a positive correlation between PSMD14 and several ERα target genes, including GREB1, TFF1, and MCM6, in breast cancer samples (Fig. 3E–G). Additionally, we analyzed the expression of PSMD14 in breast cancer samples, using IHC, and its correlation with molecular/clinical characteristics, such as ERα, PR, and HER2 status. Our results demonstrated a significant correlation between PSMD14 expression and ERα positivity (*P* < 0.001). Interestingly, PSMD14 was also positively correlated with PR expression (*P* < 0.001). We further analyzed the prognosis of PSMD14 expression in PR+ breast cancer patients. From the KMPLOT database (https://kmplot.com/analysis/index.php?p=service), we observed that PSMD14 expression correlated with shorter relapse-free survival (HR = 1.55; *P* = 0.004, Fig S3A). Besides, we carried out more cell biology assay to check if PSMD14 could regulate PR. The data showed that PSMD14 depletion not only decreased ER expression, but also decrease PR expression in both mRNA level and protein level (Fig. S3B–D). PR is known to be classical ER target gene. There is no surprise that PR expression was decreased. Furthermore, PSMD14 expression was found to correlate with lymph node metastasis and late clinical stage (*P* = 0.0139 and *P* = 0.029, respectively; Fig. 3H, I). These results indicate that PSMD14 is associated with ERα signaling activity in breast cancer.

### PSMD14 is required for ERα signaling in breast cancer

**Fig. 4:**
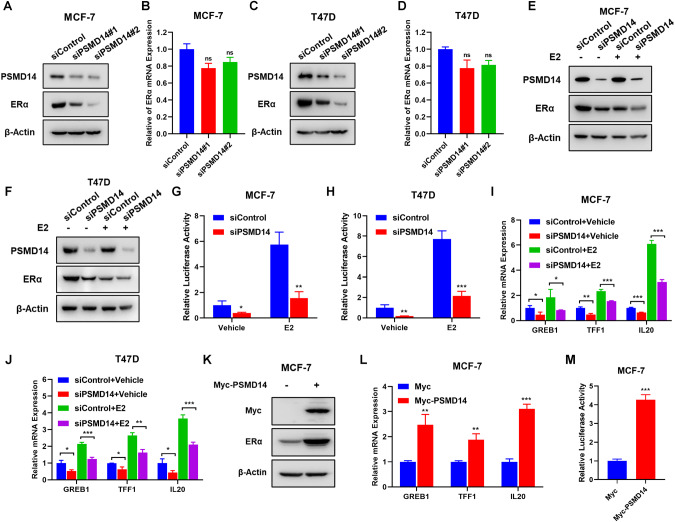
PSMD14 promotes estrogen signaling in breast cancer. **A**–**D** qRT-PCR and immunoblot analysis showing PSMD14 depletion decreases ERα protein stability but not ERα mRNA expression. MCF-7 and T47D cells were transfected with 50 nM siControl or 50 nM PSMD14. Cell lysates were immunoblotted with the indicated antibodies. β-Actin was used as internal control. **E**, **F** Immunoblot analysis showing MCF-7 and T47D cells in charcoal-stripped FBS and phenol red-free DMEM were transiently transfected with 50 nM siControl or 50 nM PSMD14 and then treated with 10 nM estradiol or vehicle for 6 h. Cell lysates were immunoblotted with the indicated antibodies. β-Actin was used as internal control. Luciferase assays showing PSMD14 depletion affects ERE-luciferase activity in MCF-7 (**G**) and T47D (**H**) cells. **I**, **J** qRT-PCR analysis of ERα target genes (GREB1, TFF1, IL20) expression in MCF-7 and T47D cells in charcoal-stripped FBS and phenol red-free DMEM were transfected with PSMD14 siRNA or Negative control for 48 h. Then treated with either ethanol or 10 nM estradiol for 6 h. Total RNA was extracted for gene expression analysis. Each group was analyzed in triplicate. *P < 0.05; **P < 0.01; ***P < 0.001 for target gene expression comparison. **K** Immunoblot analysis showing PSMD14 depletion increases ERα protein stability. MCF-7 cells were transfected with 1 μg Myc vector or Myc-PSMD14, cell lysates were immunoblotted with the indicated antibodies. β-Actin was used as internal control. **L** qRT-PCR analysis of ERα target genes (GREB1, TFF1, IL20) expression in MCF-7 showed that overexpression of PSMD14 increased the expression of ERα target genes (GREB1, TFF1, IL20). **M** Luciferase assays showing PSMD14 overexpression affects ERE-luciferase activity in MCF-7 cells. Data are shown as mean ± SD, *N* = 3. *P < 0.05; **P < 0.01; ***P < 0.001.

Since ERα plays the central role in estrogen signaling activity, we further explored the effect of PSMD14 in ERα protein. PSMD14 depletion in MCF-7 and T47D cells decreased the levels of ERα protein, but had no significant effect on the mRNA levels of ERα (Fig. 4A–D). This result suggests that PSMD14 may modulate ERα in a post-translational manner. Furthermore, when treated with estradiol, PSMD14 depletion inhibited ERα protein levels in both vehicle and estradiol-treated conditions (Fig. 4E, F). The estrogen response element luciferase assay demonstrated that PSMD14 depletion inhibited ERα signaling activity in both vehicle and estradiol-treated conditions in MCF-7 and T47D cells (Fig. 4G, H). Additionally, qRT-PCR data revealed that PSMD14 silencing inhibited the expression of ERα target genes, such as GREB1, TFF1 and IL20, in both vehicle and estradiol-treated conditions (Fig. 4I, J). Conversely, PSMD14 overexpression in MCF-7 cells increased the levels of ERα protein, ERα signaling activity, and the expression of ERα target genes, such as GREB1, TFF1 and IL20 (Fig. 4K, L). Additionally, the estrogen response element luciferase assay revealed that PSMD14 overexpression enhanced ERα signaling activity in MCF-7 cells (Fig. 4M).

### PSMD14 associates with ERα in breast cancer cells

**Fig. 5:**
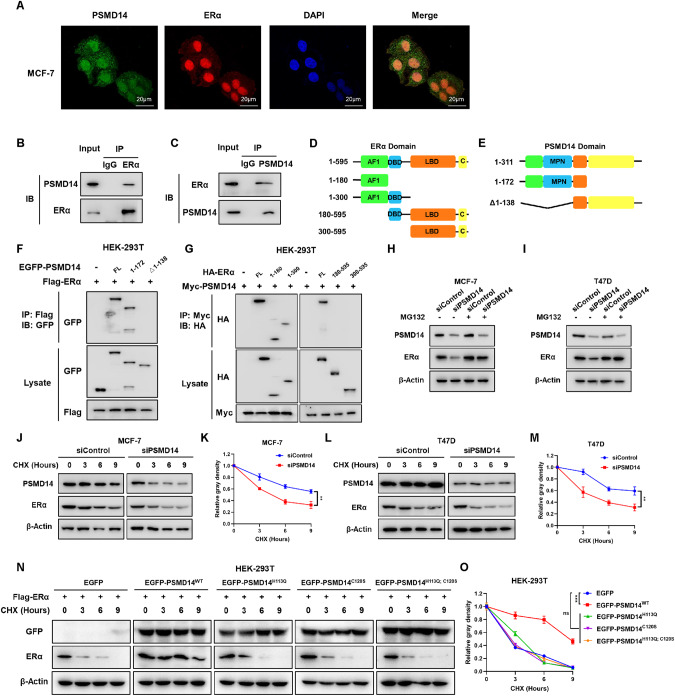
PSMD14 interacts with ERα AF1 domain through its UBD domain. **A** Immunofluorescence staining assay showing the localization patterns of PSMD14 and ERα in MCF-7 cells. Intracellular localization of PSMD14 (green) and ERα (red) is shown. Nucleus (blue) were stained with DAPI. Scale bar, 20 µm. **B**, **C** Immunoprecipitation assay showing the endogenous interaction between PSMD14 and ERα. For examining the endogenous interaction between PSMD14 and ERα, lysates of MCF-7 cells were precipitated with anti-ERα or anti-PSMD14 antibodies, and the precipitates were examined by immunoblotting. **D** Schematic of the ERα protein, along with the ERα deletion mutants (residues 1–180, 1–300, 180–595 and 300–595) used in the Co-IP assays. **E** Schematic of the PSMD14 protein, along with the PSMD14 deletion mutants (residues 1–172 and Δ1–138) used in the Co-IP assays. **F** Immunoprecipitation assay showing PSMD14 interacts with ERα through its UBD domain (1–172) which contains MPN domain (58–138). HEK-293T cells were cotransfected with 2 µg ERα plasmid and full-length GFP-PSMD14 or its mutants (1–172, Δ1–138). After 24 h, the cells were treated with 10 μM MG132 for 6 h. Then, the cells were harvested with NP-40 lysis buffer. Co-IP was performed using an anti-Flag antibody, and the possible interacting PSMD14 domains were detected with anti-GFP antibody. **G** Immunoprecipitation assay showing AF1 domain is required for ERα to interact with PSMD14. HEK-293T cells were cotransfected with 2 µg PSMD14 plasmid and full-length HA- ERα or mutant ERα (1–180, 1–300, 180–595 and 300–595). After 24 h, the cells were treated with 10 μM MG132 for 6 h. Then, the cells were harvested with NP-40 lysis buffer. Co-IP was performed using an anti-Myc antibody, and the possible interacting ERα domains were detected with anti-HA antibody. **H**, **I** Immunoblot analysis showing the expression level of ERα protein in siControl and siPSMD14 expressing MCF-7 (H) and T47D (I) cells by treat with 10 μM proteasome inhibitor MG132. **J**–**M** Immunoblot analysis showing PSMD14 increased ERα half-life. siControl and siPSMD14 expressing in MCF-7 (**J**) and T47D (**L**). Cells were treated with 100 μM cycloheximide (CHX) for the indicated times. The expression of ERα protein was estimated by ImageJ software and is represented graphically in the right panel (**K**, **M**). **N**, **O** PSMD14 deubiquitinating enzyme activity deletion mutant cannot increase ERα half-life. HEK-293T cells were cotransfected with Flag-ERα plasmid and EGFP vector or EGFP-PSMD14 WT/mutants (H113Q/C120S/H113Q; C120S) plasmid for 24 h. Then cells were treated with 100 μM cycloheximide (CHX) for the indicated times. The expression of ERα protein was estimated by ImageJ software and is represented graphically in the right panel (**O**).

We further explored the localization of PSMD14 and ERα in breast cancer cells. The immuno-staining assay showed that ERα was mainly located in the nuclear, while PSMD14 could locate in both the cytosol and nuclear (Fig. 5A). The further endogenous immuno-precipitation assay showed that PSMD14 could interact with ERα in MCF-7 cells (Fig. 5B, C). The ERα protein is composed of three functional domains: AF1 domain, DBD domain, and LBD domain (Fig. 5D). The PSMD14 protein is composed of the putative ubiquitin-binding domain (UBD) at the N-terminus, MPN domain (the catalytic domain for deubiquitinating), and the C-terminus, which contains the nuclear export signaling (NES) (Fig. 5E). To analyze the interaction between PSMD14 and ERα, we created deletion constructs. The results revealed that the UBD domain of PSMD14 is necessary for its interaction with ERα, while the interaction between PSMD14 and ERα is mediated by the AF1 domain of ERα (Fig. 5F, G). Since PSMD14 associates with ERα, the biological effect on ERα protein was further investigated. Inhibition of ERα degradation using the proteasome inhibitor MG132 showed that PSMD14 depletion decreases ERα protein level, and this effect can be reduced by MG132 treatment in MCF-7 cells (Fig. 5H, I). Additionally, the protein stability assay using the protein synthesis inhibitor cycloheximide demonstrated that PSMD14 depletion in MCF-7 and T47D cells decreases ERα protein half-life (Fig. 5J–M). Further examination of the effect of wild type and enzyme deficient forms of PSMD14 (H113Q, C120S, H113Q/C120S) on modulating ERα stability showed that the wild type form of PSMD14 can enhance ERα stability, while the catalytic deficient forms of PSMD14 are unable to do so (Fig. 5N, O). These data suggest that the effect of PSMD14 on ERα stability depends on the activity of deubiquitinase.

### PSMD14 stabilizes ERα via inhibiting ERα K48-linked poly-ubiquitination

**Fig. 6:**
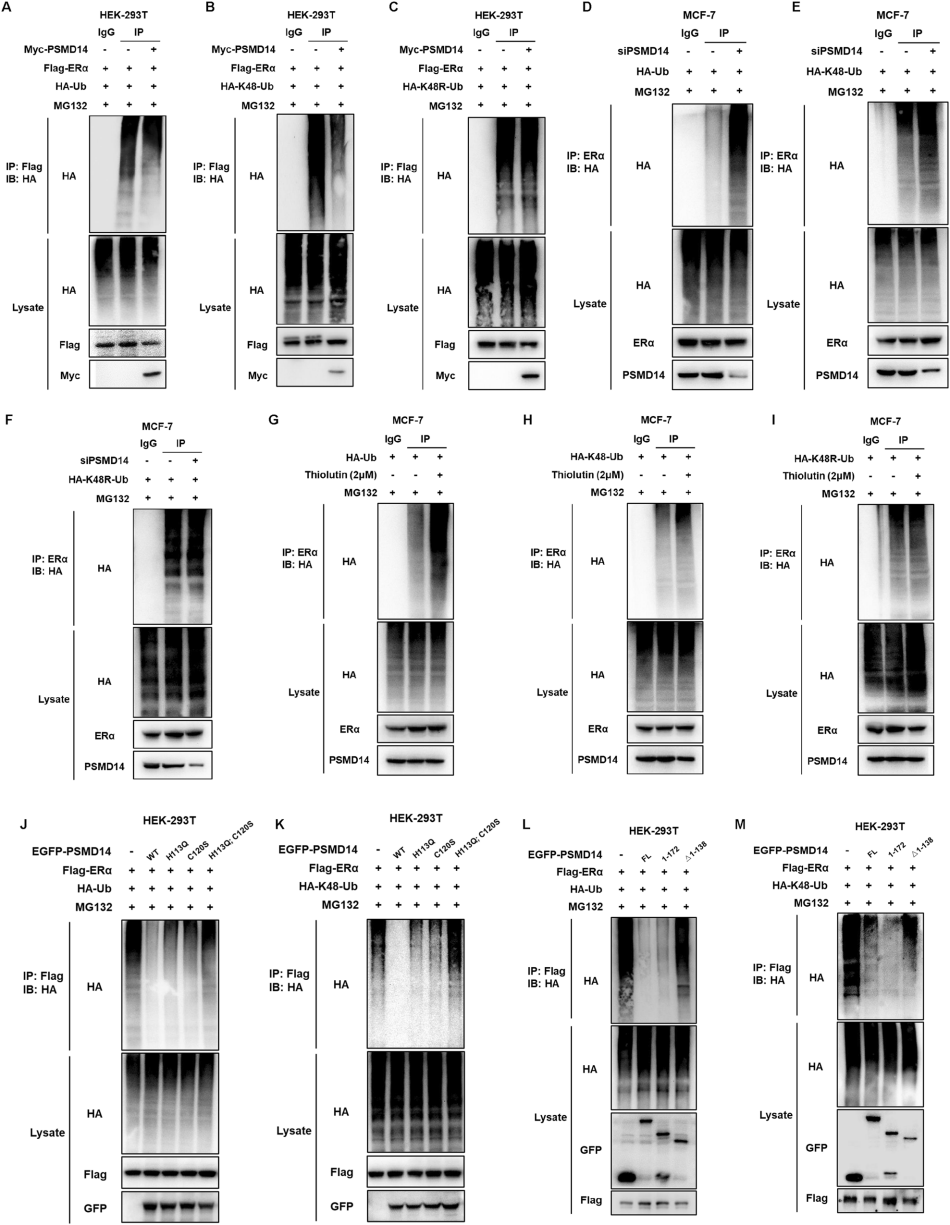
PSMD14 stabilizes ERα via inhibiting ERα K48-linked poly-ubiquitination. **A** PSMD14 reduced accumulation of polyubiquitinated ERα. HEK-293T cells were cotransfected with 2 µg ERα plasmid, 0.5 µg HA-Ub plasmid and 0.5 µg Myc-tag or Myc-PSMD14 plasmids, plasmids in HEK-293T cells upon MG132 treatment and then immunoblotted with the indicated antibodies. **B**, **C** PSMD14 Deubiquitinates ERα via K48-linked polyubiquitination. HEK-293T cells were transfected with 2 µg ERα plasmid, 0.5 µg HA-K48/HA-K48R Ub plasmid and 0.5 µg Myc-tag or Myc-PSMD14 plasmids upon MG132 treatment for 6 h and then immunoblotted with the indicated antibodies. **D** Depletion of PSMD14 increased accumulation of polyubiquitinated ERα. MCF-7 cells were transfected with 0.5 µg HA-Ub plasmid and 20uM PSMD14 siRNA upon MG132 treatment for 6 h and then immunoblotted with the indicated antibodies. **E**, **F** Depletion of PSMD14 increased ERα polyubiquitination associated with K48 but not K48R. MCF-7 cells were transfected with 0.5 µg HA-K48/HA-K48R Ub plasmid and 20 µM PSMD14 siRNA upon MG132 treatment for 6 h and then immunoblotted with the indicated antibodies. **G** PSMD14 inhibitor increased accumulation of polyubiquitinated ERα. MCF-7 cells were treated with 0.5 µg HA-Ub plasmid and 2 µM thiolutin upon MG132 treatment for 6 h and then immunoblotted with the indicated antibodies **H**, **I** PSMD14 inhibitor increased ERα polyubiquitination associated with K48 but not K48R. MCF-7 cells were treated with 0.5 µg HA-K48/HA-K48R Ub plasmid and 2 µM Thiolutin upon MG132 treatment for 6 h and then immunoblotted with the indicated antibodies. **J**, **K** PSMD14 deubiquitinating enzyme activity deletion mutant cannot increased accumulation of polyubiquitinated ERα. HEK-293T cells were transfected with 2 µg ERα plasmid, 0.5 µg HA-Ub/HA-K48 Ub plasmid and 0.5 µg EGFP-tag or EGFP PSMD14 mutants upon MG132 treatment for 6 h and then immunoblotted with the indicated antibodies **L**, **M** PSMD14 deubiquitinates ERα through its MPN domain. HEK-293T cells were transfected with 2 µg ERα plasmid, 0.5 µg HA-Ub/HA-K48 Ub plasmid and 0.5 µg EGFP-tag or EGFP PSMD14 full-length or deletion mutant plasmids upon MG132 treatment for 6 h and then immunoblotted with the indicated antibodies.

We conducted further investigations to examine the effect of PSMD14 on ERα ubiquitination. Initially, the ubiquitination-based immuno-precipitation assay was employed to demonstrate that PSMD14 has the ability to inhibit the total poly-ubiquitination level as well as the K48-linked ubiquitination level but cannot affect the K63-linked ubiquitination level of ERα in HEK-293T models (Fig. 6A, B, Fig. S4A). Moreover, it was observed that the dominant negative mutant of ubiquitin (K48R) could reduce the impact of PSMD14 on ERα poly-ubiquitination and K63R could still deubiquitinate ERα. (Fig. 6C, Fig. S4B), supporting the notion that PSMD14 specifically inhibits K48-linked ubiquitination of ERα. Subsequently, we investigated the effect of PSMD14 on ERα poly-ubiquitination in MCF-7 cells. The endogenous Co-IP assay in combination with immunoblotting for ubiquitin signals revealed that depletion of PSMD14 increased both the total poly-ubiquitination level and K48-linked ubiquitination level but cannot affect the K63-linked ubiquitination level of ERα (Fig. 6D, E, Fig. S4C). Additionally, the dominant negative mutant of ubiquitin (K48R) attenuated the effect of PSMD14 on ERα poly-ubiquitination and K63R could still deubiquitinate ERα (Fig. 6F, Fig. S4D). Considering the previous research highlighting the specific inhibitory function of thiolutin on PSMD14, we further employed thiolutin to evaluate its effects on ERα poly-ubiquitination. The endogenous Co-IP assay coupled with immunoblotting for ubiquitin signals demonstrated that pharmaceutical inhibition of PSMD14 increased both the total poly-ubiquitination level and K48-linked ubiquitination level but cannot affect the K63-linked ubiquitination level of ERα (Fig. 6G, H, Fig. S4E). Consistently, the dominant negative mutant of ubiquitin (K48R) diminished the effect of PSMD14 on ERα poly-ubiquitination and K63R could still deubiquitinate ERα (Fig. 6I, Fig. S4F). To further understand the mechanisms involved, we examined the effects of wild type and enzyme deficient forms of PSMD14 (H113Q, C120S, H113Q/C120S) on modulating ERα poly-ubiquitination, which revealed that the intact catalytic activity of PSMD14 was necessary for ERα K48-linked ubiquitination (Fig. 6J, K). Additionally, the domain-based ubiquitination assay indicated that both the intact UBD domain and MPN domain were required for PSMD14 to deubiquitinate ERα (Fig. 6L, M). The data showed that PSMD14 inhibited K48-linked poly-ubiquitination of ERα but little effect on K63-linked poly-ubiquitination of ERα.

### ERα transcriptionally regulates PSMD14 expression, which forms a forward regulation loop between ERα signaling and PSMD14

**Fig. 7:**
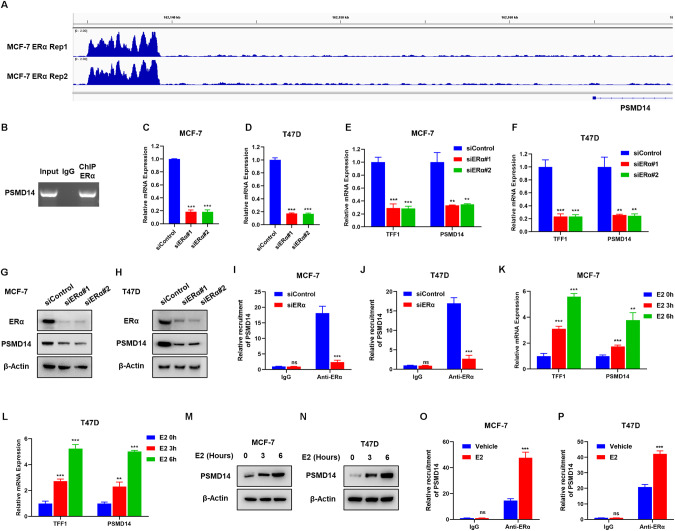
ERα transcriptionally regulates PSMD14 expression, which forms a forward regulation loop between ERα signaling and PSMD14. **A** The ChIP-seq analysis of ER binding to the PSMD14 promoter region utilized data from GEO with accession numbers GSE128208. **B** ChIP assay showed that ERα could bind to the promoter region of PSMD14. MCF-7 cells were fixed for 30 min. The Rabbit IgG was used as the negative control. The primer sequence was shown in method section. The enriched DNA fragments were subject to PCR reaction and DNA gel electrophoresis. qRT-PCR analysis showing mRNA levels of ERα after ERα depletion. MCF-7 (**C**) and T47D (**D**) cells transfected with 50 nM siControl or two independent siERα. After 48 h, total RNA was extracted for gene expression analysis. **E**, **F** ERα depletion in MCF-7 and T47D cells inhibited PSMD14 mRNA. MCF-7 and T47D cells were transfected with 50 nM siControl or siERα. After 48 h, total RNA was extracted for gene expression analysis. The relative TFF1 (positive control for ERα depletion) and PSMD14 mRNA levels were assessed by qRT-PCR. Each group was tested in triplicate. *P < 0.05; **P < 0.01; ***P < 0.001 for comparisons of target gene expression. **G**, **H** ERα depletion decreases PSMD14 protein level. MCF-7 and T47D cells were transfected 50 nM siControl or siERα. Cell lysates were immunoblotted with the indicated antibodies. β-Actin was used as internal control. **I**, **J** ChIP assay showed that ERα depletion decreases ERα recruitment to PSMD14 promoter. ERα was depleted in MCF-7 and T47D cells, and ChIP-qPCR assays showed that ERα reduced binding to PSMD14 gene. **K**, **L** E2-treated in MCF-7 and T47D cells increased PSMD14 mRNA. MCF-7 and T47D cells were treated with 10 μM E2 for the indicated times. Total RNA was extracted for gene expression analysis. The relative TFF1 (positive control for E2-treated) and PSMD14 mRNA levels were assessed by qRT-PCR. Each group was tested in triplicate. *P < 0.05; **P < 0.01; ***P < 0.001 for comparisons of target gene expression. **M**, **N** E2-treated in MCF-7 and T47D cells increased PSMD14 protein level. MCF-7 and T47D cells were treated with 10 μM E2 for the indicated times. Cell lysates were immunoblotted with the indicated antibodies. β-Actin was used as internal control. **O**, **P** ChIP assay showed that E2-treated increases ERα recruitment to PSMD14 promoter. MCF-7 and T47D cells were stimulated with E2 for 30 min and ChIP-qPCR assay showed increased binding of ERα to the PSMD14 gene. Data are shown as mean ± SD, *N* = 3. Two-tailed t test. *P < 0.05; **P < 0.01; ***P < 0.001.

Several studies have investigated the global genomic binding of ERα in breast cancer cells, as ERα plays a crucial role in modulating breast cancer progression [28, 29]. By analyzing ERα-based ChIP sequencing data [30], it was observed that there was a significant binding peak in the promoter region of PSMD14, suggesting that ERα may regulate the expression of PSMD14 (Fig. 7A). To validate this interaction, a ChIP assay confirmed the association between ERα protein and the promoter region of the PSMD14 gene (Fig. 7B). Furthermore, the depletion of ERα in breast cancer cells resulted in a decrease in PSMD14 mRNA levels (Fig. 7C–F). This decrease in ERα expression also led to a reduction in PSMD14 protein levels in both MCF-7 and T47D cells (Fig. 7G, H). To further substantiate the ERα-driven transcriptional activation of PSMD14, ERα was silenced in MCF-7 and T47D cells. The ChIP-qPCR assay confirmed that ERα depletion resulted in a decrease in ERα binding to the PSMD14 gene (Fig. 7I, J). Moreover, treatment with estradiol in MCF-7 and T47D cells increased the mRNA levels of both TFF1 and PSMD14 (Fig. 7K, L). This increase was also evident in the immuno-blotting of PSMD14 after estradiol treatment (Fig. 7M, N). Finally, the ChIP-qPCR data indicated that estradiol treatment enhanced ERα binding to the promoter region of PSMD14 in MCF-7 and T47D cells (Fig. 7O, P).

### Pharmaceutical targeting PSMD14 restrains breast cancer progression

**Fig. 8:**
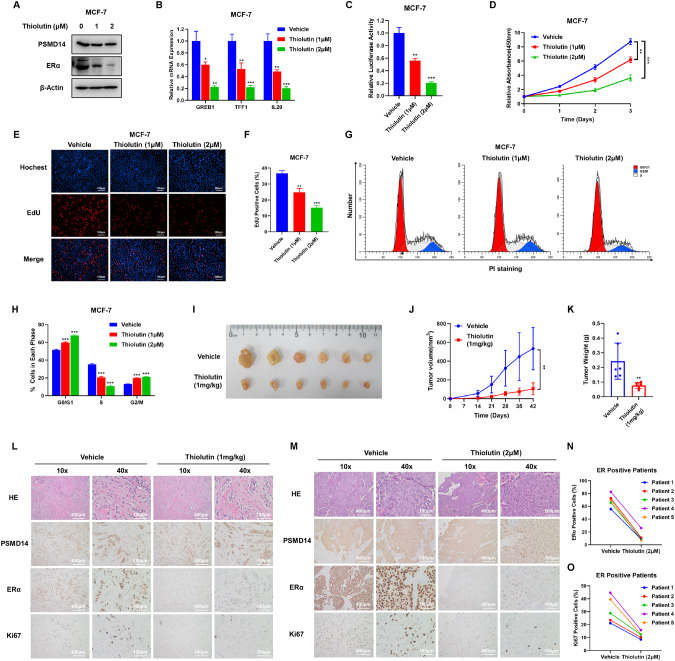
PSMD14 inhibitor Thiolutin restrains breast cancer progression. **A** Immunoblot analysis showing the PSMD14 inhibitor Thiolutin decreases ERα protein stability. MCF-7 cells were treated with different concentrations of Thiolutin. Cell lysates were immunoblotted with the indicated antibodies. β-Actin was used as internal control. **B** qRT-PCR analysis showed that the PSMD14 inhibitor Thiolutin decreases the expression of ERα target genes (GREB1, TFF1, IL20). **C** Luciferase assays showing Thiolutin affects ERE-luciferase activity in MCF-7 cells. **D** PSMD14 inhibitor Thiolutin inhibits the proliferation of ER POSITIVE breast cancer cells. MCF-7 cells were treated with different concentrations of Thiolutin. After 24 h, a CCK-8 assay was used to determine the cellular metabolic activity at the indicated time points after Thiolutin treated. Experiments were performed in triplicate. *P < 0.05; **P < 0.01; ***P < 0.001 for cell growth comparisons. **E**, **F** PSMD14 inhibitor Thiolutin reduced the number of EdU-positive ER POSITIVE breast cancer cells. MCF-7 cells were treated with different concentrations of Thiolutin. After 24 h, EdU was added to the medium for 2 h of incubation. The absolute cell number was determined to indicate cell proliferation activity (**E**). Right panel shows quantification of Edu results by ImageJ software (**F**). Scale bar 100 μm. *N* = 3, *P < 0.05; **P < 0.01; ***P < 0.001 for cell growth comparisons. **G**, **H** Cell-cycle analysis by flow cytometry of MCF-7 cells were treated with different concentrations of Thiolutin. After 24 h, the cells were harvested, fixed with 70% ethanol, and stained with propidium iodide. The cells were subjected to FACS analysis. Experiments were performed in triplicate. *P < 0.05; **P < 0.01; ***P < 0.001 for cell proportion comparisons. The PSMD14 inhibitor Thiolutin inhibits the tumor growth of MCF-7 cells in a xenograft model. Harvested and photographed tumors in the Thiolutin (1 mg/kg) and the Vehicle group (**I**), tumor volume (**J**) and weight (**K**) growth in each mouse from the Thiolition group and the Vehicle group in vivo. *P < 0.05; **P < 0.01; ***P < 0.001. **L** The levels of PSMD14, ERα and Ki67 in xenografts treated with Thiolutin by using H&E and IHC staining. Scale bar,100 µm. **M**–**O** Thiolutin inhibited cell proliferation of ER-positive breast cancer patient samples in patient-derived explant (PDEx) assay. The patient-derived tumor samples were cultured ex vivo on gelatin sponges for 48 h with 10% FBS in the presence of 2 μM Thiolutin or vehicle. The tumor samples were fixed and stained with PSMD14, ERα, and Ki67 via IHC analysis (**M**). The dynamic change of ERα positive and Ki67 positive cells were counted and shown (**N**, **O**). Scale bars are 100 μm.

Thiolutin was originally recognized as an antibiotic that specifically inhibited RNA polymerases of bacteria and yeast [31]. However, recent studies have identified thiolutin as an effective PSMD14 antagonist [24]. In order to further examine the effect of thiolutin on breast cancer phenotype and ERα signaling, we conducted various experiments. Firstly, immuno-blotting data demonstrated that thiolutin treatment reduced the protein level of ERα in MCF-7 and T47D cells (Fig. 8A, Fig S2A). qRT-PCR data also revealed that pharmaceutical inhibition of PSMD14 could suppress the expression of ERα target genes, such as GREB1, TFF1 and IL20, in both MCF-7 and T47D cells (Fig. 8B, Fig S2B). Additionally, the estrogen response element luciferase assay showed that thiolutin treatment inhibited the activity of ERα signaling in MCF-7 and T47D cells (Fig. 8C, Fig. S2C). The CCK8 assay demonstrated that thiolutin treatment significantly inhibited breast cancer proliferation (Fig. 8D, Fig. S2D). Furthermore, the EdU incorporation assay indicated that thiolutin treatment reduced the number of EdU-positive cells in MCF-7 and T47D cells (Fig. 8E, F, Fig. S2E, F). The cell cycle analysis via flow cytometry showed that thiolutin treatment significantly increased the proportion of cells in the G0/G1 phase in MCF-7 and T47D cells (Fig. 8G, H, Fig. S2G, H). To further evaluate the effect of thiolutin, we conducted an in vivo experiment using a xenograft mouse model. The results showed that thiolutin inhibited the potential of tumorigenesis in breast cancer cells, as demonstrated by the decrease in ERα and Ki67 via IHC analysis on the tumor samples (Fig. 8I–L). Utilizing an ex vivo culture model of primary breast cancer samples, we were able to evaluate the drug effect and maintain native tissue architecture. The obtained breast tumors were divided into several parts and treated with 2 μM thiolutin for in vitro growth on sponges. In the patient-derived explant assay, we found that thiolutin dramatically inhibited the level of ERα and the proliferation marker Ki67 (Fig. 8M–O).

### PSMD14 inhibition could restore tamoxifen sensitivity in endocrine resistant breast cancer model

**Fig. 9:**
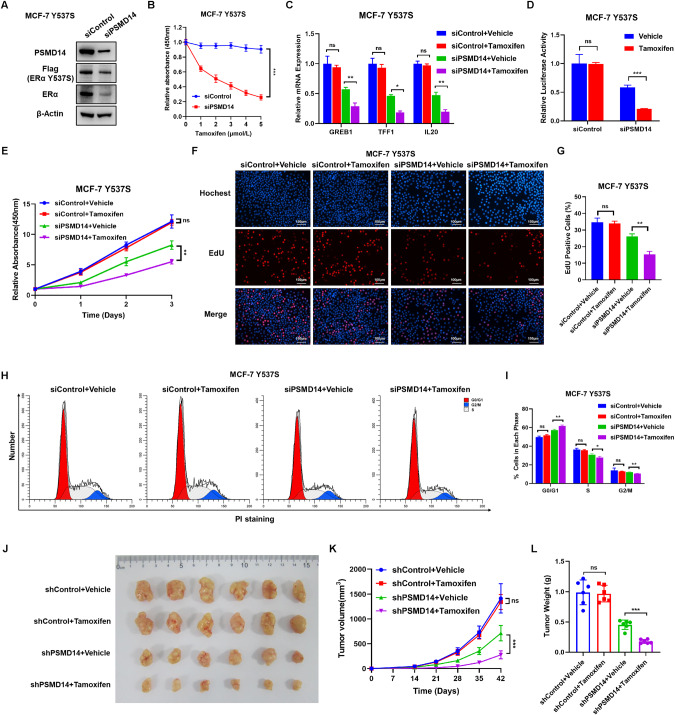
PSMD14 inhibition could restore tamoxifen sensitivity in endocrine resistant breast cancer model. **A** PSMD14 depletion decreases ERα protein level and ERα Y537S level in MCF-7 Y537S cells. Cell lysates were immunoblotted with the indicated antibodies. β-Actin was used as internal control. **B** PSMD14 depletion sensitizes tamoxifen inhibition effect in MCF-7 Y537S cells. MCF-7 Y537S cells were transfected with siPSMD14 or siControl. After 48 h, cells were plated into 96-well plate, while each well contained 5000 cells. The indicated tamoxifen concentrations were used for 48 h. The numbers of the cells were determined via CCK8 kit for the cellar metabolic activity. Experiments were done in triplicates. *P < 0.05; **P < 0.01; ***P < 0.001 for cell growth comparison. **C** PSMD14 depletion could restore the inhibition effect of tamoxifen in ERα target genes. MCF-7 Y537S cells in charcoal-stripped FBS and phenol red-free DMEM were transfected with siControl or siPSMD14 for 24 h. Then treated with 1 μM Tamoxifen for 12 h. Total RNA was extracted for gene expression analysis. Each group was analyzed in triplicate. *P < 0.05; **P < 0.01; ***P < 0.001 for target gene expression comparison. **D** Luciferase assays showing PSMD14 depletion could restore the inhibition effect of tamoxifen in ERE-luciferase activity in MCF-7 Y537S cells. **E** PSMD14 depletion could restore the inhibition effect of tamoxifen in MCF Y537S cells. MCF-7 Y537S cells in charcoal-stripped FBS and phenol red-free DMEM were transfected with siControl or siPSMD14 for 24 h. Then treated with 1 μM Tamoxifen for 12 h. Then a CCK-8 assay was used to determine the cellular metabolic activity at the indicated time points after Thiolutin treated. Experiments were performed in triplicate. *P < 0.05; **P < 0.01; ***P < 0.001 for cell growth comparisons. **F**, **G** PSMD14 depletion could reduce the number of EdU-positive cells in MCF Y537S cells. MCF-7 Y537S cells in charcoal-stripped FBS and phenol red-free DMEM were transfected with siControl or siPSMD14 for 24 h. Then treated with 1 μM Tamoxifen for 12 h. Then EdU was added to the medium for 2 h of incubation. The absolute cell number was determined to indicate cell proliferation activity. Right panel shows quantification of Edu results by ImageJ software. Scale bar 100 μm. *N* = 3, *P < 0.05; **P < 0.01; ***P < 0.001 for cell growth comparisons. **H**, **I** Cell-cycle analysis by flow cytometry of MCF-7 Y537S cells were transfected with siControl or siPSMD14 for 24 h. Then treated with 1 μM Tamoxifen for 12 h. Then the cells were harvested, fixed with 70% ethanol, and stained with propidium iodide. The cells were subjected to FACS analysis. Experiments were performed in triplicate. *P < 0.05; **P < 0.01; ***P < 0.001 for cell proportion comparisons. PSMD14 depletion could not only impair breast tumor growth but also could strengthen the inhibition effect by tamoxifen in Y537S-expression MCF-7 cells by a xenograft model. Harvested and photographed tumors in the shPSMD14 and the Control group (**J**), tumor volume (**K**) and weight (**L**) growth in each mouse from the shPSMD14 group and the Control group in vivo. Data are shown as mean ± SD. Two tailed t test. *P < 0.05; **P < 0.01; ***P < 0.001.

Here we generated the MCF-7 cell line stably expressing the mutant form of ERα (Y537S) as an endocrine resistant model. The mutations commonly found in tamoxifen-resistant breast cancer patients include Y537C/S/N in the ligand-binding domain of Erα [32]. Utilizing this model, we evaluated the effect of PSMD14 on the breast cancer phenotype and ERα signaling in an endocrine resistant background. The immuno-blotting data showed that PSMD14 depletion decreased both the wild type and mutant form of ERα (Fig. 9A). In addition, PSMD14 depletion dramatically decreased the inhibitory IC50 by tamoxifen, as observed in the IC50 assay (Fig. 9B). The qRT-PCR data indicated that PSMD14 depletion restored the inhibitory effect of tamoxifen on ERα target genes, such as GREB1, TFF1 and IL20 (Fig. 9C). PSMD14 depletion was also found to restore the inhibitory effect of tamoxifen on ERα signaling activity, as shown by the luciferase assay (Fig. 9D). The CCK8 assay further indicated that PSMD14 depletion restored the inhibitory effect of tamoxifen in breast cancer cells (Fig. 9E). This conclusion was reinforced by the results of the EdU incorporation assay and cell cycle analysis (Fig. 9F–I). Furthermore, in the xenograft mice model, PSMD14 depletion not only impaired breast tumor growth, but also enhanced the inhibitory effect of tamoxifen in the Y537S-expressing MCF-7 model (Fig. 9J–L). Hence, these findings suggest that targeting PSMD14 could overcome endocrine therapy resistance caused by mutant ERα.

## Discussion

**Fig. 10:**
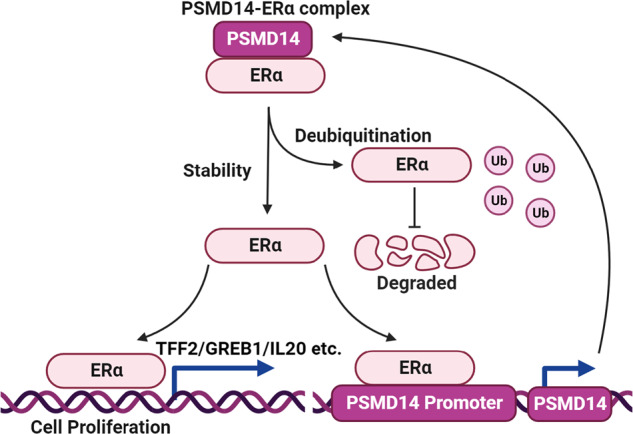
A hypothetical model of the mechanism of PSMD14 regulation of ERα signaling and the positive feedback loop formed by PSMD14 and ERα in breast cancer. The activation PSMD14 facilitates breast cancer progression via modulating ERα K48-linked deubiquitinating, which subsequently enhanced ERα signaling activity. Besides, ERα could directly induce the expression of PSMD14, which implicated a positive feedback loop between PSMD14 and ERα signaling.

We found that PSMD14 is correlated with the gene signature of ERα signaling and associated with poor survival in luminal type breast cancer. We discovered that PSMD14 facilitates breast cancer progression by modulating ERα K48-linked deubiquitinating, thereby enhancing ERα signaling activity. Furthermore, we found evidence that ERα directly induces the expression of PSMD14, suggesting a positive feedback loop between PSMD14 and ERα signaling (Fig. 10). This novel positive feedback loop reveals a non-genomic regulation mechanism for ERα signaling and highlights PSMD14 as a potential therapeutic target for breast cancer treatments.

The success of endocrine therapy in breast cancer is the hallmark for cancer research [33]. The selective estrogen receptor modulators, such as tamoxifen, have been applied in the clinics for more than 30 years [34, 35]. Currently the luminal type of breast cancer achieves the longest survival among all human malignancies [36]. However, the endocrine resistance could be induced with long time endocrine therapy. According to recent statistics, about 30% of endocrine therapy patients could be primary resistance [33], while 40% endocrine therapy patients are acquired resistance [37]. The endocrine resistant patients could only achieve approximately 3 years of survival. Thus, it is urgent and necessary to uncover the potential mechanism and novel therapeutic targets, which could enhance endocrine therapy efficacy or reverse endocrine resistance. Quite a lot of efforts were taken to discover the possible mechanisms. For example, several studies have shown a few co-activators or transcriptional co-factors could synergize with ERα signaling to overcome tamoxifen inhibition, such as FOXA1 (Forkhead Box A1) [38]. Besides, several AP1 (Activation Protein 1) were also found to cooperate with ERα signaling to mediate tamoxifen resistance [39, 40]. However, there are no mature drugs targeting such transcriptional factors. Based on the fact that most of the resistant mechanisms are still dependent on ERα function. Targeting ERα could be a promising strategy to overcome endocrine resistance in breast cancer [41].

In recent studies, more and more E3 ubiquitin ligases have been shown to affect ERα signaling function and tamoxifen sensitivity [42]. For example, our previous studies showed that RNF181 and SMURF1 could associate with ERα and promote breast cancer proliferation [43, 44]. Besides, some other studies showed EFP or BRCA1 could induce ERα ubiquitination and degradation [45]. Although the effects on ERα protein could be diverse, all these studies might indicate the conclusion that the ubiquitination status of ERα is an important modification, which subsequently affect ERα signaling function and endocrine therapy outcome. Since ERα function is subject to a tiny balance between E3 ubiquitin ligases and deubiquitinases, inhibition of certain deubiquitinases expression or function, which subsequently lead to enhance ERα poly-ubiquitination and degradation, could be an effective strategy for breast cancer treatment [46]. In our study, our DUB screening data revealed PSMD14 was a critical factor, which could be a useful drug target for breast cancer.

PSMD14 (Proteasome 26S Subunit, Non-ATPase 14) was firstly discovered as the component of 26 S proteasome non-ATP subunit, which also belongs to JAMM metalloprotease family [16, 20]. In human malignancies, PSMD14 was over-expressed in liver cancer, esophageal carcinoma and breast cancer, which expression also related to poor overall survival [47]. As one oncogene, PSMD14 was shown to deubiquinating several substrates to promote cancer progression [25]. For example, PSMD14 could deubiquitinate snail protein and promote esophageal cancer cell EMT (Epithelial Mesenchymal Transition) [24, 27]. Besides, PSMD14 could also stabilize E2F1, which enhanced E2F1 target gene expression and cancer progression in head and neck squamous carcinoma [48, 49]. However, the oncogenic mechanism of PSMD14 in breast cancer is not totally clear. Our studies revealed a novel functional link between PSMD14 and ERα. PSMD14 could be a novel drug target for breast cancer treatment.

In conclusion, we proved PSMD14 as an oncogene for breast cancer both in clinical samples and experimental studies. We illustrated that the expression of PSMD14 was increased in breast cancer and related to poor survival only in luminal type breast cancer patients. PSMD14 interacted with ERα protein, inhibited ERα poly-ubiquitination and proteasome-dependent degradation in breast cancer cells. Our studies revealed a novel function of PSMD14 in estrogen signaling in multiple layers. As a novel modulator for estrogen signaling, modulation of PSMD14 activity or gene expression level could be an appealing strategy to treat breast cancer. Although we uncover the regulatory feedback loop between ERα and PSMD14 in luminal type breast cancer, there are several limitations of our study. Firstly, the regulation mechanism was based on several ER+ cancer cell lines, while it is worthwhile to identify in vivo whether genetic depletion of PSMD14 in mammary gland could regulate breast epithelial cells and subsequently carcinogenic process of breast cancer in transgenic mouse models. Besides, due to the difficulties to acquire purified PSMD14 protein, we failed to perform pulldown assay to show the direct interactions between PSMD14 and ERα. Thus, the further biochemical and structural biology work could be carried out for cocrystal structure of PSMD14-ERα for drug development.

## Materials and methods

### Cell lines and cell culture

The human breast cancer MCF-7, T47D, MDA-MB-175, and HEK-293T cells were purchased from American Type Culture Collection (ATCC). Cell line authentication was performed via short tandem repeat (STR) using the Promega Power Plex 21 system. These cell lines were maintained in Dulbecco’s Modified Eagle’s Medium (DMEM, D6429, Sigma-Aldrich), supplemented with 10% Fetal Bovine Serum (FBS, 10270-106, Gibco), 1% penicillin–streptomycin–gentamicin Solution (C0223, Beyotime), 4.5 g/L glucose, 4 mM l-glutamine, and incubated at 37 °C with 5% CO_2_. For E2 assays, cells were cultured in charcoal-stripped FBS (Gibco, 12676-029) treated with phenol red-free DMEM (Gibco, 21063029) and then supplemented with 17b-estradiol (E2; Sigma-Aldrich) dissolved in ethanol.

To generate lentiviruses for PSMD14 depletion, short hairpin RNA (shRNA) lentiviral particles against PSMD14 were transduced into HEK-293T cells, following the manufacturer’s protocol. The transfection was conducted by co-transfecting the cells with pMD2G and psPAX_2_ envelop plasmids. The lentivirus was obtained after 2 days of transfection. T47D (ERα positive breast cancer cells) were incubated with 2 mL antibiotic-free medium containing 200 μL lentivirus. To generate stable cell lines, infected cells were selected using 2 μg/mL puromycin (Merck Millipore).

### Plasmids and siRNA

The Myc-PSMD14 plasmid, EGFP-PSMD14 plasmid, HA-ERα and Flag-ERα plasmid were acquired from Origene Company (https://www.origene.com). HA-Ub, HA-K48, HA-K48R, HA-K63 and HA-K63R plasmids were used in previous study. The Lipofectamine 2000 (1662298, Invitrogen) was used for the transfection of plasmids. We used the small interfering RNAs to knockdown the specific gene. The PSMD14 siRNA sequences were siRNA#1: GGC AUU AAU UCA UGG ACU ATT; siRNA#2: GAU GGU UGU UGG UUG GUA UTT; Negative control: UUC UCC GAA CGU GUC ACG UTT. The RNA iMAX reagent (13778150, invitrogen) was used for the transfection of siRNA. The PSMD14 shRNA sequences used were showed as following: GCA GCA GAA CAA GTC TAT AT; Negative control: UUC UCC GAA CGU GUC ACG U.

### DUB siRNA library screening

The siRNA library consisting of 76 human DUBs was purchased from Human Deubiquitinating Enzyme (ON-TARGET plus) from Dharmacon siRNA Library, GU-104705). ERα positive breast cancer cells were transfected with different siDUBs in MCF-7. After transfection for 48 h, RNA was extracted, and RNA was reverse transcribed into cDNA. The levels of the ERα classical downstream gene, TFF1, were measured to screen for significant regulation of the ERα signaling pathway by the results of this study focused on the following Using the preliminary results, this study focused on the deubiquitinating enzyme PSMD14.

### Real-time quantitative PCR (qRT-PCR)

We used Rneasy Puls Mini Kit (Qiagen, China; Cat: 4992235) to extract total RNA and reverse-transcribed into cDNA using Revert Aid First Strand cDNA Synthesis Kit (Thermo, Lithuania), and then amplified by PCR using specific primers and SYBR Green (A25742, Thermo Fisher) on a 7500 real-time fluorescence quantitative PCR system (Applied Biosystems, Singapore) with the expression of 36B4 as the internal reference. Primer sequences are shown in Supplementary Table S1.

### Western blotting

Cells were lysed with RIPA buffer (Beyotime). Protein samples were loaded on to and separated using SDS/PAGE, transferred on to PVDF membranes blocked and probed with the primary antibodies. After blocking the membranes for 1 h at room temperature in 5% skim milk powder dissolved in Tris-buffered saline containing 5% Tween-20 (TBST), membranes were incubated overnight at 4 °C with the corresponding antibodies. Then Membranes were washed by PBST for three times and probed with secondary antibodies. After washing, the blots visualized using the ECL system (Bio-rad ChemiDoc).

### Luciferase assay

The cells were transfected with 50 nM PSMD14 siRNA or 1 µg Myc-PSMD14 plasmids, the luciferase reporter and the renilla plasmid. About 24 h later, Cells were harvested, and Luciferase activity was measured. The luciferase activity of ERα were monitored using the Dual Luciferase Reporter kit (Promega).

### CCK8 assay

Twenty-four hours after silencing PSMD14, both the silenced group and control group were plated onto 96-well plates with 4000 cells per well at 24, 48, and 72 h. Cell counts were determined at these time points using the CCK8 absorbance assay.

### EdU assay

Cell proliferation was determined by EdU (5-ethynyl-20-deoxyuridine) assay using the EdU Cell Proliferation Assay Kit (Ribobio, Guangzhou, China). MCF-7, T47D and MDA-MB-175 cells were spread into 96-well plates after corresponding treatment. After 24 h, cells were added with 50 mM EdU and continued to incubate for 2 h. Then fix the cells with 4% paraformaldehyde and stain with proliferating cells using Apollo dye solution. Nucleic acid was stained with Hoechst 33342. The cell proliferation rate was calculated using the imageJ.

### Flow cytometric analyses

MCF-7, T47D and MDA-MB-175 cells were treated with Thiolutin for cell cycle analysis. For siPSMD14 knockdown experiments, MCF-7 cells were transfected with siPSMD14 or siControl. Similarly, for MCF-7 Y537S cells, they were treated with siPSMD14 or Tamoxifen. After 24 h, the treated cells were digested and resuspended into a single cell suspension, followed by washing in PBS. Ethanol was used to fix the cells, which were subsequently stained with propidium iodide. Fluorescence intensity was measured using BD LSR flow cytometry.

### Co-Immunoprecipitation assay

Cell proteins were collected with Western and IP lysates, adding proteasome inhibitor (ST506 P0013, Beyotime). 12,000 × *g* at 4 °C after centrifugation for 30 min, the collected supernatant was incubated with the required antibody or control IgG and protein A/G agarose (P2051/P2053, Beyotime) at 4 °C overnight. The next day at 4 °C, 3000 × *g* after centrifugation for 10 min, rinse with lysis buffer (P0013F, Beyotime) for three times and discard the supernatant. Then add 2× SDS-PAGE buffer, 99 °C boiling 10 min, and going on immunblotting.

### Protein stability assay

Cycloheximide (CHX) chase assay was used to determine the half-life of endogenous or ectopically expressing ERα. Cells were cultured in 12-well plates, with about 105 cells per well, and were transfected with 50 nM siControl/siPSMD14 or 1 µg vector/EGFP-PSMD14 WT/EGFP-PSMD14 Mutants. After 48 h, the cells were treated with 100 µM cycloheximide (C7698, Sigma) for the indicated time points. Subsequently, equal amounts of boiled lysates were analyzed by immunoblotting western blotting to detect ERα degradation.

### Poly-ubiquitination detection assay

K48-linked poly-ubiquitination as an example. To directly detect K48-linked poly-ubiquitination of ERα from cell extracts, the cells were co-transfected with 2 µg of Myc-PSMD14 or Myc tagged with 0.5 µg of K48 Ubi plasmids and 0.5 µg of Flag ERα for 24 h. After that, the cells were treated with 10 μM MG132 for 6 h. Protein extraction was performed and then subjected to preclearance with 30 µL of protein A (P2051, Beyotime) for 2 h. Next, the extract was incubated overnight with anti-ERα antibody or anti-FLAG antibody, followed by incubation with protein A/G beads for 1 h at 4 °C. Finally, the western blotting technique was employed to detect total polyubiquitinated ERα or K48-polyubiquitinated ERα using anti-HA antibody.

### Immunofluorescence assay

MCF-7 cells were fixed with 4% paraformaldehyde (p0099, Beyotime) for 10 min, permeabilized with PBS containing 0.2% Triton X-100 (T8200, Solarbio) for 10 min at room temperature. And then blocked by PBS plus 5% BSA (ST025, Beyotime) for 1 h. Rabbit anti-PSMD14 polyclonal antibody (HPA002114, SIGMA) and mouse anti-ERα Monoclonal antibodies (SC-56833, Santa Cruz) were used overnight at 4 °C, followed by Alexa flow 647 (Invitrogen) anti rabbit antibody and FITC coupled anti mouse antibody (Jackson ImmunoResearch, West Grove, PA) for 1 h at room temperature in dark. After three times washing, acquiring a final concentration of 0.1 μg/ml DAPI (Sigma) to stain nucleus. The images were captured by (acquired with) confocal laser scanning microscope. The collected images are further processed and analyzed by ImageJ.

### Clinical breast tumor samples

One hundred and sixteen breast cancer specimens were collected from the pathology department of Qilu Hospital, Shandong University. The PSMD14 status, ER alpha status, PR status, and HER2 status of all breast tumor samples were examined by pathologists. Pathological specialists also examined the pathological grading and lymph node metastasis status of each sample. The study of clinical samples, which had received written informed consent from all the patients, was reviewed and approved by the Ethical Board at Shandong University.

### Publicly available clinical data analysis

PSMD14 tumor RNA-seq data in breast cancer can be obtained from the Genomic Data Commons (GDC) data portal website (https://portal.gdc.cancer.gov/). The acquired data were analyzed and calculated by Prism 8.0 (GraphPad). Analysis of PSMD14 correlation with ERα target genes (MCM6, TFF1, and GREB1) was carried out by the Cancer Genome Atlas (TCGA), database using 879 breast cancer samples. PSMD14 expression analysis of ER-positive, HER2-positive, triple-negative breast cancer tissues, and normal tissues, as well as the expression of individual breast cancer stages, was conducted by the TCGA database (https://www.genome.gov/Funded-Programs-Projects/Cancer-Genome-Atlas). Analysis of the association of PSMD14 expression with clinical prognosis was implemented using the KMPLOT database (https://kmplot.com).

### Computational analysis of RNA sequencing data

Gene set enrichment analysis (GSEA) was performed using the GSEA program provided by the Broad Institute (http://www.broadinstitute.org/gsea/index.jsp). GSEA was used to assess the relative enrichment of ERα positive regulated genes in two different groups, siControl and siPSMD14. The enrichment analysis utilized Hallmark gene sets and KEGG pathways by Metascape (https://metascape.org), which allowed for exploration of pathways associated with differentially expressed genes (DEGs). Additionally, the OmicStudio tools (https://www.omicstudio.cn/tool) were used to provide a volcano plot of the DEGs, with a threshold of *P* < 0.05 and fold change > 1.5.

### Chromatin immunoprecipitation (ChIP) assay

The ChIP assay was conducted to analyze MCF-7 and T47D cells. The cells were fixed for 30 min to enable cross-linking. Following fixation, a mixture of 0.1375 mol/L glycine was added to the cells for neutralization. The cells were then washed with cold PBS/1 mmol/L PMSF and subsequently scraped into PBS/1 mmol/L PMSF, followed by centrifugation. After centrifugation, the cell pellet was treated with SDS lysis buffer. Next, the cells were sonicated for 10 min (30 s on/off) to fragment the chromatin. The ChIP assay kit (Millipore, 17-295) was utilized for the subsequent steps of the assay. In the ChIP experiments, the anti-ERα rabbit antibody (D8H8, #8644) was employed. Quantitative PCR analysis was performed with a DNA extraction kit (Qiagen, Cat. No. 28106). Primer sequences for ChIP-qPCR are displayed here: PSMD14 F: GGG GGC ACG CTA GAA TAA ACT, R: CAA CGC AGC CCT GTT TTG AA; TFF1 F: GGG CTT CAT GAG CTC CTT C, R: TTC ATA GTG AGA GAT GGC CGG; The ChIP-seq analysis of ER binding to the PSMD14 promoter region shown in Fig. 7A utilized data from GEO with accession numbers GSE128208.

### Xenograft mouse models

In vivo tumorigenic experiment, we used the 5-week-old female BALB/c nude mice which were purchased from Shanghai Model Organisms Center, inc. Slow-release 17 beta-estradiol pellets (0.72 mg/90 days, Innovative Research of America) were implanted into the mice. After 24 h, 4 × 10^6^ cancer cells were injected into the mammary fat pad of each mouse, using 150 μL of PBS. Tumor formation in the mice was then monitored for approximately 6 weeks. The tumor volume was calculated using the formula: tumor volume = length × width^2^/2. In Thiolutin treatment experiments, mice were randomly assigned to experimental groups containing 6 mice each. Thiolutin was injected intraperitoneally at a dosage of 1 mg/kg per day for one month, while the control group received solvent injections. All experimental procedures involving mice were conducted in accordance with the guidelines approved by the Xin Xiang Medical University Animal Care Commission.

### Patient-derived explant (PDEx) assay

The excised tissues for research purposes, approved by the ethical committee of Qilu Hospital, Shandong University, were processed according to the ex vivo culture protocol. In general, breast cancer tissues were cultured on gelatin sponges with the cell culture medium containing 10% FBS. The tissues were either treated with vehicle or Thiolutin for 48 h. Subsequently, the tissues were fixed in 10% formaldehyde at 4 °C overnight. To confirm the quality, the tissues were stained with hematoxylin and eosin. After that, the immunohistochemistry was performed to examine the indicated markers.

### Statistics

Student’s *t*-test, Pearson correlation coefficient, and Cox regression analysis were Publicly available data. Data are expressed as the mean ± SEM. Differences were considered to be statistically significant when *P* < 0.05. *P < 0.05; **P < 0.01; ***P < 0.001.

### Supplementary information

Supplementary Figure Legends

Supplementary Figure 1

Supplementary Figure 2

Supplementary Figure 3

Supplementary Figure 4

Supplementary Table 1

## Data Availability

The publicly available data are provided in GEO database (GSE197078 and GSE128208). The original siRNA screening data are provided in supplementary materials. The original data for WB and qRT-PCR are provided in supplementary materials. The cell line authentications are shown in supplementary materials.
