# Supplementary material for: PSMD14 stabilizes estrogen signaling and facilitates breast cancer progression via deubiquitinating ERα
Source: Oncogene. 2023 Nov 29;43(4):248–64. doi: 10.1038/s41388-023-02905-1 (PMC10798890; doi:10.1038/s41388-023-02905-1)
Supplement: Supplementary file 1 — Supplementary Figure Legends [file 41388_2023_2905_MOESM1_ESM.docx]

**Supplementary Figure Legends**

**Supplementary Figure 1: PSMD14 depletion inhibits cell proliferation in T47D and MDA-MB-175 cells.**

**A-D:** qRT-PCR and immunoblot analysis showing the expression level of PSMD14 in T47D and MDA-MB-175 cells transfected with siControl or two independent siPSMD14. Cell lysates were immunoblotted with the indicated antibodies. β-Actin was used as the internal control.

**E-F:** PSMD14 depletion inhibits the proliferation of ER POSITIVE breast cancer cells. T47D and MDA-MB-175 cells were transfected with 50 nM siControl or 50 nM PSMD14. After 24 h, a CCK-8 assay was used to determine the cellular metabolic activity at the indicated time points after transfection. Experiments were performed in triplicate. **P* < 0.05; ***P* < 0.01; ****P* < 0.001 for cell growth comparisons.

**G-J**: PSMD14 depletion reduced the number of EdU-positive ER positive breast cancer cells. T47D and MDA-MB-175 cells were transfected with 50 nM siControl or 50 nM PSMD14. After 24 h, EdU was added to the medium for 2 h of incubation. The absolute cell number was determined to indicate cell proliferation activity (G, I). Right panel shows quantification of EdU results by ImageJ software (H, J). Scale bar 100 μm. N=3, **P* < 0.05; ***P* < 0.01; ****P* < 0.001 for cell growth comparisons.

**K-N:** Cell-cycle analysis by flow cytometry of T47D and MDA-MB-175 cells transfected with 50 nM siControl or 50 nM PSMD14. After 24 h, the cells were harvested, fixed with 70% ethanol, and stained with propidium iodide. The cells were subjected to FACS analysis. Experiments were performed in triplicate. **P* < 0.05; ***P* < 0.01; ****P* < 0.001 for cell proportion comparisons.

**Supplementary Figure 2: PSMD14 inhibitor Thiolutin restrains breast cancer progression in T47D cell line.**

**A:** Immunoblot analysis showing the PSMD14 inhibitor Thiolutin decreases ERα protein stability. T47D cells were treated with different concentrations of Thiolutin. Cell lysates were immunoblotted with the indicated antibodies. β-Actin was used as internal control.

**B:** qRT-PCR analysis showed that the PSMD14 inhibitor Thiolutin decreases the expression of ERα target genes (GREB1, TFF1, IL20).

**C:** Luciferase assays showing Thiolutin affects ERE-luciferase activity in T47D cells.

**D:** PSMD14 inhibitor Thiolutin inhibits the proliferation of ER POSITIVE breast cancer cells. T47D cells were treated with different concentrations of Thiolutin. After 24 h, a CCK-8 assay was used to determine the cellular metabolic activity at the indicated time points after Thiolutin treated. Experiments were performed in triplicate. **P*< 0.05; ***P* < 0.01; ****P*< 0.001 for cell growth comparisons.

**E-F:** PSMD14 inhibitor Thiolutin reduced the number of EdU-positive ER POSITIVE breast cancer cells. T47D cells were treated with different concentrations of Thiolutin. After 24 h, EdU was added to the medium for 2 h of incubation. The absolute cell number was determined to indicate cell proliferation activity (E). Right panel shows quantification of Edu results by ImageJ software (F). Scale bar 100 μm. N=3, **P* < 0.05; ***P* < 0.01; ****P* < 0.001 for cell growth comparisons.

**G-H:** Cell-cycle analysis by flow cytometry of T47D cells were treated with different concentrations of Thiolutin. After 24 h, the cells were harvested, fixed with 70% ethanol, and stained with propidium iodide. The cells were subjected to FACS analysis. Experiments were performed in triplicate. **P*< 0.05; ***P* < 0.01; ****P*< 0.001 for cell proportion comparisons.

**Supplementary Figure 3: PSMD14 regulates PR and affects prognosis in PR+ breast cancer patients**

**A:** Kaplan-Meier analysis showing relapse-free survival depending on PSMD14 expression levels from public meta-analysis data (https://kmplot.com). PSMD14 expression was correlated with poor survival in PR positive human breast cancer. *P* values were calculated using log-rank test (HR=1.55; *P*=0.004).

**B-D**: qRT-PCR and immunoblot analysis showing PSMD14 depletion decreases PR-A/B protein stability and PR-A/B mRNA expression. T47D cells were transfected with 50 nM siControl or 50 nM PSMD14. Cell lysates were immunoblotted with the indicated antibodies. β-Actin was used as internal control.

**Supplementary Figure 4: PSMD14 does not deubiquitinate ERα via K63-linked polyubiquitination.**

**A-B**: PSMD14 does not deubiquitinate ERα via K63-linked polyubiquitination. HEK-293T cells were transfected with 2 µg ERα plasmid, 0.5 µg HA-K63/HA-K63R Ub plasmid and 0.5 µg Myc-tag or Myc-PSMD14 plasmids upon MG132 treatment for 6 hours and then immunoblotted with the indicated antibodies.

**C-D**: Depletion of PSMD14 cannot affect ERα K63-linked ubiquitination. MCF-7 cells were transfected with 0.5 µg HA-K63/HA-K63R Ub plasmid and 20 µM PSMD14 siRNA upon MG132 treatment for 6 hours and then immunoblotted with the indicated antibodies.

**E-F**: PSMD14 inhibitor cannot affect ERα K63-linked ubiquitination. MCF-7 cells were treated with 0.5 µg HA-K63/HA-K63R Ub plasmid and 2 µM Thiolutin upon MG132 treatment for 6 hours and then immunoblotted with the indicated antibodies.

**Supplementary Table 1: The sequence and antibody list**

Antibody and primer sequences for qRT-PCR and siRNA/shRNA sequences for PSMD14 depletion are listed in Supplementary Table 1
