# Supplementary figures and images for: PSMD14 stabilizes estrogen signaling and facilitates breast cancer progression via deubiquitinating ERα
Source: Oncogene. 2023 Nov 29;43(4):248–64. doi: 10.1038/s41388-023-02905-1 (PMC10798890; doi:10.1038/s41388-023-02905-1)

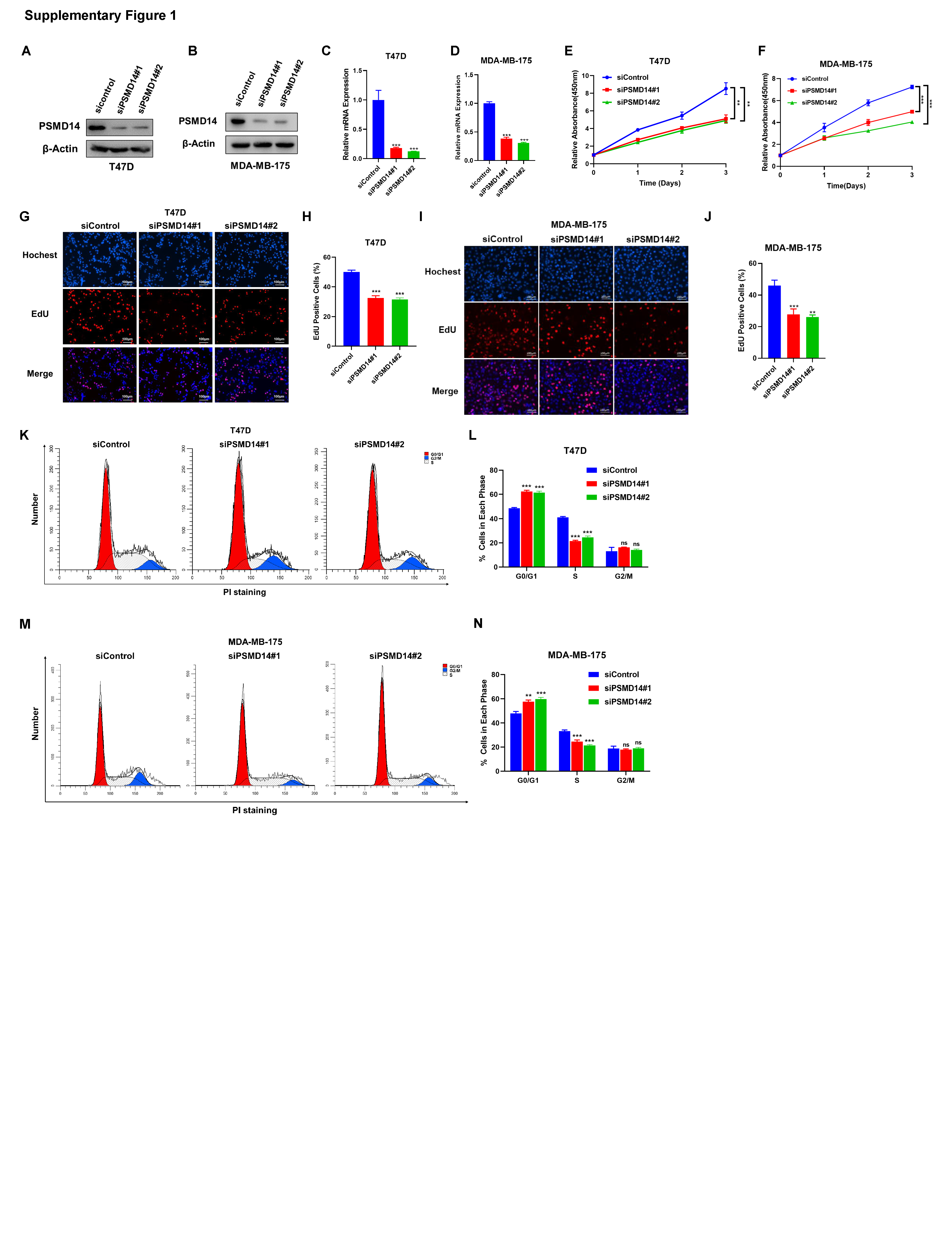

Supplement: Supplementary file 2 — Supplementary Figure 1 [file 41388_2023_2905_MOESM2_ESM.tif]

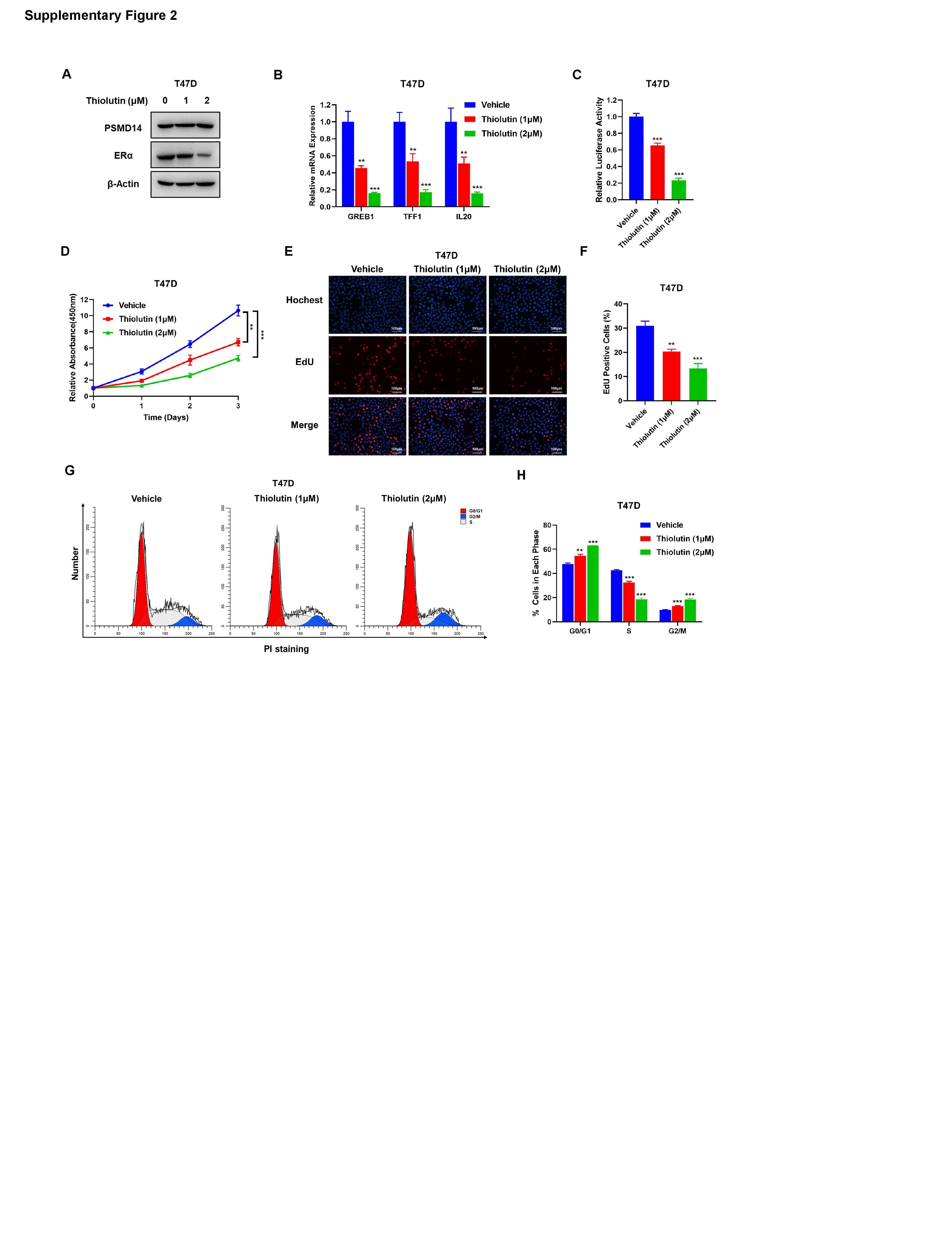

Supplement: Supplementary file 3 — Supplementary Figure 2 [file 41388_2023_2905_MOESM3_ESM.tif]

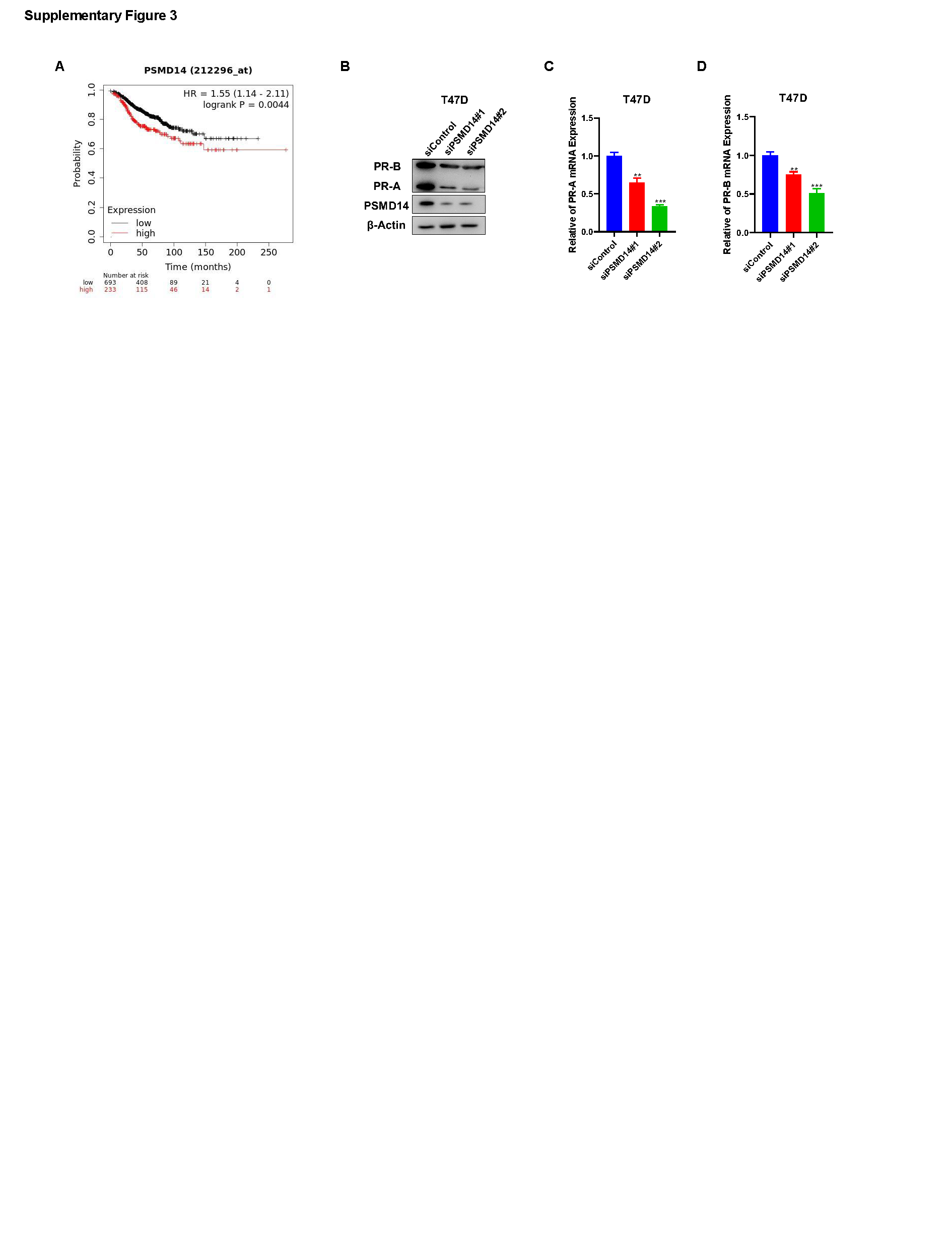

Supplement: Supplementary file 4 — Supplementary Figure 3 [file 41388_2023_2905_MOESM4_ESM.tif]

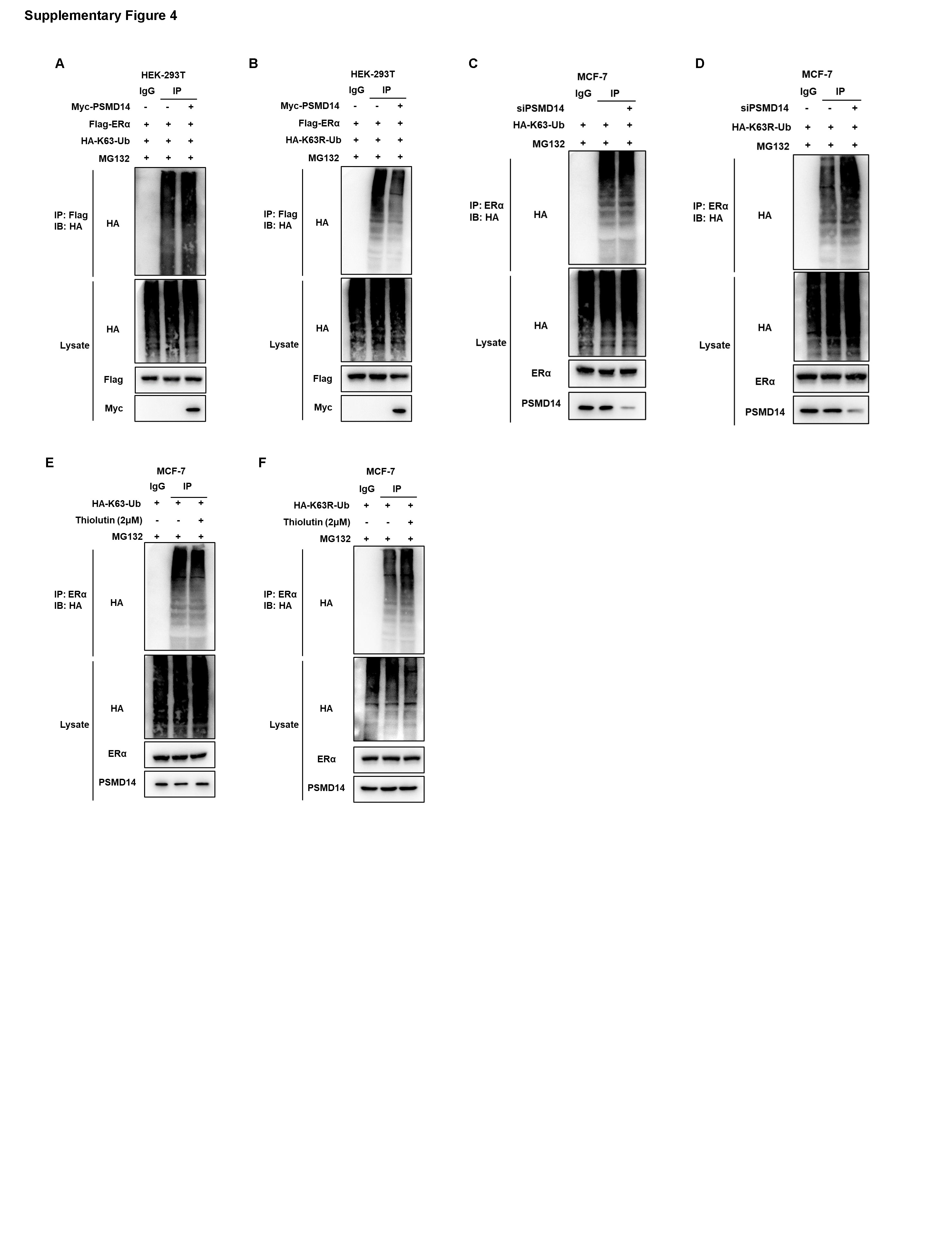

Supplement: Supplementary file 5 — Supplementary Figure 4 [file 41388_2023_2905_MOESM5_ESM.tif]
